# Four New Glycosides from the Rhizoma of *Anemarrhena asphodeloides*

**DOI:** 10.3390/molecules22111995

**Published:** 2017-11-22

**Authors:** Bing-You Yang, Xue-Yan Bi, Yan Liu, Guo-Yu Li, Xin Yin, Hai-Xue Kuang

**Affiliations:** 1 Key Laboratory of Chinese Materia Medica, Ministry of Education of Heilongjiang, University of Chinese Medicine, Harbin 150040, China; ybywater@163.com (B.-Y.Y.); lifeliuyan@163.com (Y.L.); yinxin110901@163.com (X.Y.); 2 Heilongjiang Institute for Food and Drug Control, Harbin 150001, China; hljbixueyan@sina.cn; 3 College of Pharmacy, Harbin University of Commerce, Harbin 150001, China; Leegy@163.com

**Keywords:** *Asparagaceae*, natural product, pyroglutamic acid, disaccharide, steroidal saponins, cytotoxicity

## Abstract

Four new compounds, aneglycoside A–C (**1**–**3**) and timosaponin U (**4**), were isolated from the rhizomes of *Anemarrhena asphodeloides*. Their structures were determined through extensive spectroscopic analysis, chemical characteristics, and high-resolution mass spectrometry (HRMS). All the isolations were evaluated for cytotoxicity against HepG2, Hela, and SGC7901 human cancer lines. Compounds **1**, **2**, and **4** showed weak antiproliferative activities on HepG2, Hela, and SGC7901 cells.

## 1. Introduction

**Figure 1:**
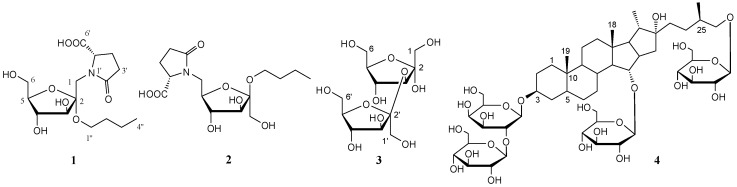
Structures of compounds **1**–**4** from *Anemarrhena asphodeloides*.

*Anemarrhena asphodeloides* Bunge (*Asparagaceae*) is a perennial herb, widely distributed in China, particularly in the Hebei and Anhui provinces. The dried rhizomes of *A. asphodeloidesis* is a commonly used traditional Chinese medicine known as “Zhimu”, used for its heat-clearing, fire-purging, Yin-nourishing, and dryness-moistening effects [1]. Steroidal saponins, flavonoids, and alkaloids are the major components of *A. asphodeloidesis* [2], resulting in various biological functions, including anti-tumor, anti-oxidant, anti-inflammation, anti-hypertension, and anti-hyperglycemic properties [3,4]. During further investigation of the bioactive constituents, four new compounds were found from the *n*-butanol layer of *A. asphodeloidesis* aqueous extract, including two new pyroglutamic acid-fructosides, aneglycoside A (**1**) and aneglycoside B (**2**), one new disaccharide, aneglycoside C (**3**), and one new steroidal saponin (**4**) (Figure 1). In this paper, we reported the isolations and structures of the new compounds **1**–**4**, as well as their cytotoxicity activities.

## 2. Experimental Method

### 2.1. General Experimental Procedures

Optical rotations were measured on a JASCO P-2000 instrument (JASCO, Tokyo, Japan). The infrared (IR) spectra were recorded on a Shimadzu FTIR-8400S (Shimadzu, Tokyo, Japan). High-resolution electrospray ionization mass spectrometry (HRESIMS) was conducted using a Waters Xevo-TOF-MS™ instrument (Waters, Bedford, MA, USA). The ultraviolet (UV) spectra were recorded on a UV-2450 spectrometer (Shimadzu, Kyoto, Japan). Semi-preparative and preparative high-performance liquid chromatography (HPLC) was performed on an Agilent 1100 liquid chromatography (Agilent Corporation, Waldbronn, Germany) with a Zorbax SB-C18 (9.4 mm × 25 cm) column (Agilent Corporation, Palo Alto, CA, USA). The hydrogen (^1^H), carbon (^13^C), and two-dimensional (2D) (^1^H-^1^H correlation spectroscopy (COSY), heteronuclear multiple bond correlation (HMBC), heteronuclear multiple quantum coherence (HSQC)) nuclear magnetic resonance (NMR) spectra were recorded on a JNM-ECA600 spectrometer (JEOL, Tokyo, Japan) using a standard pulse sequence. Column chromatography was performed using silica gel (100–120 mesh and 200–300 mesh, Qingdao Marine Chemical Co., Qingdao, China). The thin-layer chromatography used GF_254_ (MN), and spots were detected by spraying the plates with 10% H_2_SO_4_-EtOH reagent followed by heating at 105 °C for 5 min. 

### 2.2. Plant Material

The rhizomes of *A. asphodeloides* were collected from the Anhui Province in China in April 2015, and identified by Ruifeng Fan of Heilongjiang University of Chinese Medicine. A voucher specimen (S 2015100803) was deposited at the Heilongjiang University of Chinese Medicine.

### 2.3. Extration and Isolation

Air-dried *A. asphodeloides* rhizomes (20 kg) were extracted under reflux with three times the amount of water for 2 h, then the residue was filtered, and extracted with seven times the amount of purified water for 1 h, then this process was repeated. The filtrates were combined and evaporated to a suitable concentration. Then, 95% ethanol was added four or five times to adjust the concentration of ethanol to 80%, allowed to stand for 1 day, filtered in vacuo, and centrifuged. The filtrate was concentrated in a vacuum to eliminate ethanol, and then purified with macroporous resin. The 95% ethanol fraction was evaporated in vacuo followed by suspension in water. The aqueous layer was further partitioned with ethyl acetate and *n*-butanol. The *n*-butanol-soluble was evaporated under reduced pressure to result in a residue of 751 g, which was chromatographed on a silica gel column of MeOH-CH_2_Cl_2_ (9:1, 5:1, 1:1 *v*/*v*) to create five fractions. Fraction 1 was fractioned into nine subfractions (Fractions 1.1–1.9) using a silica gel column eluted with CH_2_Cl_2_-MeOH (6:1). Fraction 1.7 was further separated by preparative HPLC MeOHåH_2_O (30:70) to yield compound **1** and **2**. Fraction 5 was subjected to octadecylsilyl (ODS) chromatography (H_2_O/MeOH, 1:0 to 0:1) to create six subfractions. Fraction 5.1 was further separated by preparative HPLC MeOH-H_2_O (5:95) to yield compound **3**. Fraction 4 was subjected to ODS chromatography (H_2_O/MeOH, 1:0 to 0:1) to create sevben subfrations. Fraction 7.5 was further separated by preparative HPLC MeOH-H_2_O (50:50) to yield compound **4**.

#### 2.3.1. Aneglycoside A—Compound **1**

White amorphous powder. ${\left[\alpha \right]}_{D}^{25}$ −23.7 (c = 2.1, MeOH); ^1^H- and ^13^C-NMR (MeOH, 400, 100 MHz) data are shown in Table 1, Figures S1 and S2; high-resolution electrospray ionization mass spectrometry (HR-ESI-MS) *m*/*z* 348.1661 [M + H]^+^ (calcd. for C_15_H_26_NO_8_, 348.1658).

#### 2.3.2. Aneglycoside B—Compound **2**

White amorphous powder. ${\left[\alpha \right]}_{D}^{25}$ −26.4 (c = 1.9, MeOH); ^1^H- and ^13^C-NMR (MeOH, 400, 100 MHz) data; see Table 1, Figures S3 and S4; HR-ESI-MS *m*/*z* 348.1643 [M + H]^+^ (calcd. for C_15_H_26_NO_8_, 348.1658).

#### 2.3.3. Aneglycoside C—Compound **3**

White amorphous powder. ${\left[\alpha \right]}_{D}^{25}$ +19.8 (c = 1.7, D_2_O); ^1^H- and ^13^C-NMR (MeOH, 400, 100 MHz) data; see Table 2, Figures S5 and S6; HR-ESI-MS *m*/*z* 343.1203 [M + H]^+^ (calcd. for C_12_H_23_O_11_, 343.1240).

#### 2.3.4. Timosaponin U—Compound **4**

White amorphous powder. ${\left[\alpha \right]}_{D}^{25}$ −43.2 (c = 1.4, MeOH); ^1^H- and ^13^C-NMR (MeOH, 400, 100 MHz) data, shown in Table 3, Figures S7 and S8; HR-ESI-MS *m*/*z* 1099.5528 [M + H]^+^ (calcd. for C_51_H_87_O_25_, 1099.5536).

### 2.4. Acid Hydrolysis, GC Analysis, and Opitcal Rotation Test

Compounds **1**–**4** (2.0 mg) were refluxed with HCl (2 mol/L, 5 mL) for 4 h at 90 °C. Then, the reagent was neutralized with sodium hydroxide (NaHCO_3_), and extracted with 5 mL methanol (MeOH) four times. The remaining aqueous layer was concentrated each time and then freeze-dried to provide a residue. The residue was dissolved in 1 mL pyridine and 0.7 mL silylation-derived agent, and added to the solution for shaking for 5 min, then placed at room temperature for 4 h, and 1.5 mL distilled water was added. After centrifugation, the supernatant was detected by gas chromatography (GC) [5,6]. The glycosyl configurations of compounds **1**–**4** were determined by the same retention time (*t*_R_) of standard d-fructose (*t*_R_ = 6.3 min) for compounds **1**–**3**, and d-galactose and d-glucose for compound **4** (*t*_R_ = 32.5 min and *t*_R_ = 16.8 min). Meanwhile, the MeOH extract layer was concentrated by rotary evaporation and then the optical rotation (OR) test was conducted. The configurations of pyroglutamic acid in compounds **1** and **2** were determined by the same [α] of standard l-pyroglutamic acid (${\left[\alpha \right]}_{D}^{25}$ −27.5 (c = 10, NaOH)) [7].

### 2.5. Cytotoxicity Assays

The isolations were evaluated for their antiproliferative activities using the 3-(4,5-dimethylthiazol-2-yl)-2,5-diphenyl-2-*H*-tetrazolium bromide (MTT) method in vitro on HepG2, Hela, and SGC7901 cells obtained from the Shanghai Institute of Biochemistry and Cell Biology. They were cultured in Roswell Park Memorial Institute (RPMI) 1640 (10% Fetal bovine serum, 100 IU/mL penicillin, and 100 μg/mL streptomycin) in 5% CO_2_ at 37 °C. Then, the cells were cultured in 96-well plates for 24 h with 100 μL complete medium, and the compounds were added with varying concentrations of 5.0, 10.0, 25.0, 50.0 and 100 μg/mL. MTT (20 μL) with 5 mg/mL phosphate buffer saline was added for another 4 h in the 96-well plates, then dissolved in dimethyl sulfoxide (DMSO) and assayed at 490 nm by the VICTOR-X3 ELISA instrument (PerkinElmer, Massachusetts, USA) [6]. The cytotoxicity of the compounds against HepG2, Hela, and SGC7901 was calculated and expressed as an IC_50_ value. Doxorubicin was used as the positive control (Table 4). 

## 3. Results

### 3.1. Structure Determination

**Figure 2:**
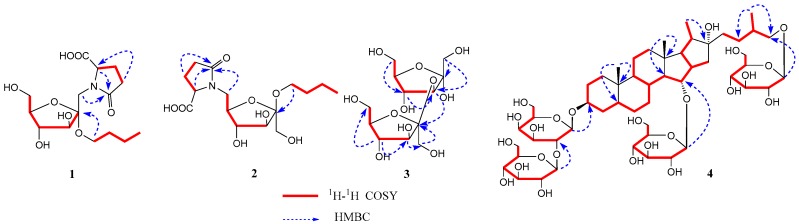
Key heteronuclear multiple bond correlation (HMBC) and ^1^H-^1^H correlation spectroscopy (COSY) correlations of compounds **1**–**4**.

Compound **1** was isolated as a white powder. Its molecular formula C_15_H_25_NO_8_ was determined from data of the positive-ion HRESIMS (*m*/*z*: 348.1661 [M + H]^+^, (calcd. 348.1658). The ^1^H-NMR spectrum of compound **1** (Table 1) showed the signals of δ_H_ 4.32 (1H, dd, *J* = 8.0, 4.0 Hz, –CH), 2.49 (1H, m, –CH_2_-a), 2.33 (2H, m, –CH_2_), and 2.23 (1H, m, –CH_2_-b), assigned to l-pyroglutamic acid protons. Additionally, a group of the signals of d-fructose was observed at δ_H_ 4.35 (1H, d, *J* = 11.6 Hz, *–*OCH_2_-1a), 4.24 (1H, d, *J* = 11.6 Hz, –OCH_2_-1b), 3.96 (1H, d, *J* = 8.0 Hz, H-3), 3.91 (1H, d, *J* = 8.0 Hz, H-4), 3.73 (1H, o, H-6a), 3.71 (1H, o, H-5), and 3.56 (1H, o, H-6b). A group of *n*-butoxyl moiety proton signals were found at δ_H_ 3.74 (1H, m, –OCH_2_-a), 3.52 (1H, m, –OCH_2_-b), 1.53 (2H, m, –CH_2_), 1.37 (2H, m, –CH_2_), and 0.92 (3H, t, *J* = 7.2 Hz, –CH_3_). The ^13^C-NMR spectrum also exhibited 15 carbon signals, in three groups of carbon signals (δ_C_ 102.9, 83.5, 80.2, 76.8, 64.7, and 64.0; δ_C_ 62.3, 33.2, 20.3, and 14.3; δ_C_ 181.2, 173.4, 57.1, 30.2 and 25.8), attributed to d-fructose, *n*-butoxyl moiety, and l-pyroglutamic acid, respectively. Six carbon signals (δ_C_ 102.9, 83.5, 80.2, 76.8, 64.7 and 64.0) were similar to those of the sugar moiety of α-d-fructofuranoside [4]. Furthermore, glutamic acid that appeared in the form of l-pyroglutamic acid was corroborated by the HMBC of the correlation of δ_H_ 4.32 (1H, dd, *J* = 8.0, 4.0 Hz, pyr-H-5) with carbon signals at δ_C_ 181.2 (C-6′) and 173.4 (C-2′). The sugar linkage sites were determined by the HMBC correlation (Figure 2) between δ_H_ 4.35 (1H, d, *J* = 11.6 Hz, Fruc-H-1-a), 4.24 (1H, d, *J* = 11.6 Hz, Fruc-H-1-b), and δ_C_ 173.4 (pyr-C-2′), suggesting that pyroglutamic acid was linked by N to form a glycoside with C-1 of the fructosyl moiety. In addition, the correlation between δ_H_ 3.74 (1H, m, *n*-butoxyl-OCH_2_-a) and δ_C_ 102.9 (Fruc-C-2) indicated that the *n*-butoxyl group was linked to the fructosyl moiety at C-2. Additionally, the absolute configurations of fructofuranose and pyroglutamic acid were determined as d- and l-configurations by the acid hydrolysis, GC analysis, and OR test. From the above data and prior research [7,8], the structure of compound **1** was identified as 1-deoxy-1-[l-pyroglutamic acid]-2-*n*-butoxy-α-d-furanofructoside, named aneglycoside A. 

Compound **2** was a white powder. Its molecular formula C_15_H_25_NO_8_ was deduced from the data of the positive-ion HRESIMS at *m*/*z* 348.1643 [M + H]^+^ (calcd. 348.1658). The ^1^H- and ^13^C-NMR data (Table 1) indicated that compound **2** was an *n*-butyl-(pyroglutamic acid) six-carbon sacchroside. By comparing the ^1^H-NMR and ^13^C-NMR spectra data of compounds **1** and **2**, the configuration of the fructosyl moiety was found to represent the difference. The ^13^C-NMR data of the sugar moiety of compound **2** were similar to those of β-d-fructofuranoside [4]. Comparing the ^13^C-NMR spectrum data of compound **2** with that of fructose, fruc-C-6 was approximately up-field shifted by 3.0 ppm. The HMBC correlation (Figure 2), between δ_H_ 4.28 (2H, o, Fruc-H-6) and δ_C_ 173.4 (pyr-C-2′), suggested that pyroglutamic acid was linked by N to form a glycoside with C-6 of the fructosyl moiety. Furthermore, the correlation between δ_H_ 3.61 (1H, m, *n*-butoxyl-OCH_2_-a) and δ_C_ 105.6 (Fruc-C-2) indicated that the *n*-butoxyl group was linked to the fructosyl group at C-2. Like compound **1**, the absolute configuration of fructofuranose and pyroglutamic acid in compound **2** was determined as d- and l-configuration. Therefore, the structure of compound **2** was established as 6-deoxy-6-[l-pyroglutamic acid]-2-*n*-butoxy-α-d-furanofructoside, and named aneglycoside B. 

Compound **3** was a white powder, and its molecular formula was C_12_H_22_O_11_ according to HRESIMS at *m*/*z* 343.1203 [M + H]^+^ (calcd. 343.1240). The ^13^C-NMR (Table 2) spectrum of compound **3** showed the two characteristic signals of anomeric carbon at δ_C_ 98.8 and δ_C_ 102.5, which were deduced to be a disaccharide. Combined with the Distortionless Enhancement by Polarization Transfer (DEPT), the ^13^C-NMR spectrum of compound **3** showed 12 signals, in two groups of fructofuranosyl at δ_C_ 98.8, 81.8, 77.7, 83.5, 61.2, and 61.7, and δ_C_ 62.5, 102.5, 76.9, 74.5, 81.2 and 62.6. The α-configuration of fructofuranosyl was determined by the chemical shift of C-3 (δ_C_ 81.8) and C-5 (δ_C_ 83.5), and the β-configuration of fructofuranosyl was determined by the chemical shift of C-5′ (δ_C_ 81.2) based on the literature [9]. The connectivity of the two fructofuranosyls was mainly established on the HMBC correlation of H-3 (δ_H_ 3.94, d, *J* = 2.4 Hz) with C-2′ (δ_C_ 102.5) (Figure 2), which suggested the attachment position of β-fructofuranosyl at C-2 of α-fructofuranosyl. The absolute configuration of fructofuranosyl groups of compound **3** was determined as D and D by GC analysis after derivatization. Thus, the structure of compound **3** was determined to be α-d-fructofuranosyl-(3→2)-β-d-fructofuranose, and named aneglycoside C.

Compound **4** was isolated as a white amorphous powder, with the molecular formula of C_51_H_86_O_25_ determined by HRESIMS at *m*/*z* 1099.5528 [M + H]^+^ (calcd. for 1099.5536). The ^1^H-NMR spectrum of compound **4** (Table 3) showed four methyl proton signals of a typical steroidal skeleton at δ_H_ 1.03 (3H, d, *J* = 6.7 Hz, H_3_-27), δ_H_ 1.32 (3H, d, *J* = 6.8 Hz, H_3_-21), δ_H_ 0.89, and 0.95 (3H each, both s, H_3_-19, 18). In the ^13^C-NMR spectrum of compound **4** (Table 3), four methyl groups (δ_C_ 18.2, 24.3, 16.6, and 17.6) and quaternary carbon (δ_C_ 110.5) suggested that compound **4** was a furostanol saponin. The ^13^C-NMR data of compound **4** were similar to those of timosaponin E1 [10], obtained previously from the rhizomes of *Anemarrhena asphodeloides*. The major difference between them was that an additional signal of glucose in compound **4** was missing in timosaponin E1. The glucose linkage was established by the existence of long-range HMBC correlations between δ_H_ 4.80 (H-1′′′) and δ_C_ 79.2 (C-15), suggesting the attachment position of the glucose at C-15. Assignments of all groups of compound **4** were achieved through ^1^H-^1^H COSY, HSQC, and HMBC (Figure 2). The absolute configuration of the glycosyls group of compound **4** was determined by GC analysis after derivatization. The nuclear overhauser effect (NOESY) correlation of Me-19/H-1β, H-5; H-1α/H-3, Me-18/H-15, indicated the α-orientation of H-3 and β-orientation of H-15. According to the literature [11], the absolute configuration of C-25 was determined by the gap between two hydrogen protons (∆^ab^) in C-26. The absolute configuration of C-25 in compound **4** was *R*, based on the ∆^ab^ = 0.45 gap (∆^ab^ ≤ 0.48 was *R*, ∆^ab^ ≥ 0.59 was *S*). The absolute configuration of glucopyranosyl groups of compound **4** was determined as D by GC analysis. Synthesizing the above results, the structure of compound **4** was determined to be (25*R*)-15-*O*-β-d-glucopyranosyl-26-*O*-β-d-glucopyranosyl-22- hydroxy-5β-furost-3β,15α,26-diol-3-*O*-β-d-glucopyranosyl(1→2)-β-d-galactopyranoside, and named timosaponin U.

### 3.2. Cytotoxic Activity

The compounds **1**–**4** were evaluated against three human tumor cell lines (HepG2, Hela, and SGC7901) for their cytotoxic activities using the MTT method. The research results showed that aneglycoside A and B (compounds **1** and **2**) showed weak cytotoxicity against HepG2 and Hela cells with an IC_50_ value of 38.4, 29.7, and 41.7, 34.2 μM, respectively. Timosaponin U (compound **4**) exhibited weak cytotoxicity against HepG2, Hela, and SGC7901 cells with IC_50_ values of 61.8, 39.7, and 44.5 μM, respectively. However, compound **3** displayed no cytotoxicity activity against these three human tumor cells, and compounds **1** and **2** did not show significant cytotoxicity activity on SGC7901 (Table 4).

## 4. Conclusions

*A. asphodeloidesis* possesses many kinds of bioactivities, such as anti-oxidant, anti-tumor, anti-inflammation, and blood sugar reduction capabilities, and has been applied to the treatment of febrile diseases with high fever. This study obtained four new glycosides compounds from *A. asphodeloidesis*, including pyroglutamic acid fructosides and steroidal saponins, and their cytotoxicity activities were evaluated. These results indicated these glycoside compounds could be the pharmacodynamic material basis for *A. asphodeloidesis*. Further study on the chemical constituents of *A. asphodeloidesis* could contribute to discovering active ingredients and leading compounds, and provide an experimental and scientific basis for drug design and drug discovery.

## Figures and Tables

**Table 1:** ^1^H-nuclear magnetic resonance (NMR) and ^13^C-NMR data for compounds **1** and **2** (CD_3_OD).

No.	Compound 1		Compound 2	
	δ_C_	δ_H_ Mult (*J*, Hz)	δ_C_	δ_H_ Mult (*J*, Hz)
**Sugar**				
**1**	64.0	4.35 (d, 11.6)	61.7	3.65 (d, 11.8)
		4.24 (d, 11.6)		3.52 (d, 11.8)
**2**	102.9		105.6	
**3**	80.2	3.96 (d, 8.0)	78.1	4.12 (d, 8.0)
**4**	76.8	3.91 (d, 8.0)	77.4	3.99 (d, 8.0)
**5**	83.5	3.71 overlap	80.0	3.92 overlap
**6**	64.7	3.73 overlap	67.5	4.28 overlap
		3.56 (m)		
**Pyroglutamic Acid**				
**2′**	173.4		173.8	
**3′**	25.8	2.49 (m)	25.9	2.49 (m)
		2.23 (m)		2.21 (m)
**4′**	30.2	2.33 (m)	30.2	2.34 (m)
**5′**	57.1	4.32 (dd, 8.0, 4.0)	57.0	4.30 (m)
**6′**	181.2		181.1	
***n*-butyl**				
**1″**	62.3	3.74 (m)	62.3	3.61 (m)
		3.52 (m)		3.45 (m)
**2″**	33.2	1.53 (m)	33.4	1.52 (m)
**3″**	20.3	1.37 (m)	20.3	1.37 (m)
**4″**	14.3	0.92 (3H, t, 7.2)	14.4	0.92 (3H, t, 7.2)

**Table 2:** The ^1^H- and ^13^C-NMR data of compound **3** (D_2_O).

No.	δ_C_	δ_H_ Mult (*J*, Hz)	No.	δ_C_	δ_H_ Mult (*J*, Hz)
α-Fruf			β-Fruf		
**1**	98.8	3.60 (d, 12.3)	**1′**	62.5	3.52 (d, 12.3)
		4.13 (d, 12.3)			4.06 (d, 12.3)
**2**	81.8		**2′**	102.5	
**3**	77.7	3.94 (d, 2.4)	**3′**	76.9	3.67 (d, 7.8)
**4**	83.5	3.86 (dd, 2.4, 5.7)	**4′**	74.5	4.02 (d, 7.8)
**5**	61.2	3.92 (m)	**5′**	81.2	3.83 (m)
**6**	61.7	3.68 (d, 12.3)	**6′**	62.6	3.58 (d, 12.4)
		3.77 (d, 12.3)			3.77 (d, 12.4)

**Table 3:** ^1^H- and ^13^C-NMR data for compound **4** (C_5_D_5_N).

No.	δ_C_	δ_H_ Mult (*J*, Hz)	No.	δ_C_	δ_H_ Mult (*J*, Hz)
**1**	31.2	1.86 (m)	**3-gal**		
		1.48 (m)	**1′**	102.4	4.89 (d, 7.6)
**2**	27.2	1.84 (m)	**2′**	81.6	4.65 (m)
		1.29 (m)	**3′**	76.9	4.03 (m)
**3**	75.5	4.32 (m)	**4′**	69.9	4.54 (m)
**4**	31.0	1.48 (m)	**5′**	76.6	4.07 (m)
		1.85 (m)	**6′**	62.2	2.06 (m)
**5**	37.0	2.15 (m)			4.42 (m)
**6**	27.1	1.92 (m)	**glc**		
		1.52 (m)	**1″**	106.0	5.27 (d, 7.6)
**7**	27.0	1.87 (m)	**2″**	75.3	4.37 (m)
		1.28 (m)	**3″**	78.1	4.18 (m)
**8**	36.5	1.85 (m)	**4″**	71.8	4.27 (m)
**9**	40.9	1.21 (m)	**5″**	78.4	3.83 (m)
**10**	35.5		**6″**	62.8	4.52 (m)
**11**	21.3	1.26 (m)			4.32 (m)
		1.37 (m)	**15-glc**		
**12**	41.5	1.21 (m)	**1′′′**	105.1	4.80 (d, 7.8)
		1.69 (m)	**2′′′**	75.3	4.27 (m)
**13**	41.4		**3′′′**	78.5	3.82 (m)
**14**	61.0	1.62 (m)	**4′′′**	71.8	4.18 (m)
**15**	79.2	4.38 (m)	**5′′′**	78.6	3.95 (m)
**16**	91.4	5.06 (dd, 3.6, 8.7)	**6′′′**	62.8	4.52 (m)
**17**	61.5	2.25 (m)			4.32 (m)
**18**	18.2	0.95 (3H, s)	**26-glc**		
**19**	24.3	0.89 (3H, s)	**1′′′′**	105.2	4.83 (d, 7.8)
**20**	40.5	1.42 (m)	**2′′′′**	75.3	4.27 (m)
**21**	16.6	1.32 (3H, d, 6.8)	**3′′′′**	78.7	4.19 (m)
**22**	110.5		**4′′′′**	71.8	4.23 (m)
**23**	37.2	1.98 (m)	**5′′′′**	78.6	3.95 (m)
		2.12 (m)	**6′′′′**	62.9	4.53 (m)
**24**	28.5	1.72 (m)			4.36 (m)
		2.09 (m)			
**25**	34.5	1.92 (m)			
**26**	75.1	3.55 (m)			
		4.00 (m)			
**27**	17.6	1.03 (3H, d, 6.7)			

**Table 4:** Cytotoxicity of compounds **1**–**4**.

Compound	IC_50_ (μM)			Compound	IC_50_ (μM)		
	HepG2	Hela	SGC7901		HepG2	Hela	SGC7901
**1**	38.4 ± 2.4	29.7 ± 1.9	>100	**4**	61.8 ± 4.1	39.7 ± 3.7	44.5 ± 2.0
**2**	41.8 ± 3.5	34.2 ± 3.6	>100	doxorubicin	8.4 ± 2.2	9.0 ± 1.4	6.7 ± 1.8
**3**	>100	>100	>100				

## Supplementary Materials

Supplementary Materials are available online.
