# Supplementary material for: Four New Glycosides from the Rhizoma of Anemarrhena asphodeloides
Source: Molecules. 2017 Nov 22;22(11):1995. doi: 10.3390/molecules22111995 (PMC6150167; doi:10.3390/molecules22111995)

Supporting Information

Four New Glycosides from the Rhizoma of *Anemarrhena asphodeloides*

Bing-You Yang [1,†], Xue-Yan Bi [2,†], Yan Liu [1], Guo-Yu Li [1], Xin Yin [1] and Hai-Xue Kuang [1,*]

[1] Key Laboratory of Chinese Materia Medica, Ministry of Education of Heilongjiang University of Chinese Medicine, Harbin 150040, China; ybywater@163.com (B.-Y.Y.); lifeliuyan@163.com (Y.L.);
Leegy@163.com (G.L.); yinxin110901@163.com (X.Y.)

[2] Heilongjiang Institute for Food and Drug Control, Harbin 150001, China; hljbixueyan@sina.cn

* Correspondence: hxkuang@hotmail.com; Tel.: +86-451-8219-7188

† Bing-You Yang and Xue-Yan Bi have contributed equally to this work.

Caption:

Fig.S1. ^{1}H-NMR Spectrum of Compound **1**

Fig.S2. ^{13}C-NMR Spectrum of Compound **1**

Fig.S3. ^{1}H-NMR Spectrum of Compound **2**

Fig.S4. ^{13}C-NMR Spectrum of Compound **2**

Fig.S5. ^{1}H-NMR Spectrum of Compound **3**

Fig.S6. ^{13}C-NMR Spectrum of Compound **3**

Fig.S7. ^{1}H-NMR Spectrum of Compound **4**

Fig.S8. ^{13}C-NMR Spectrum of Compound **4**

Fig.S1. ^{1}H-NMR Spectrum of Compound **1**

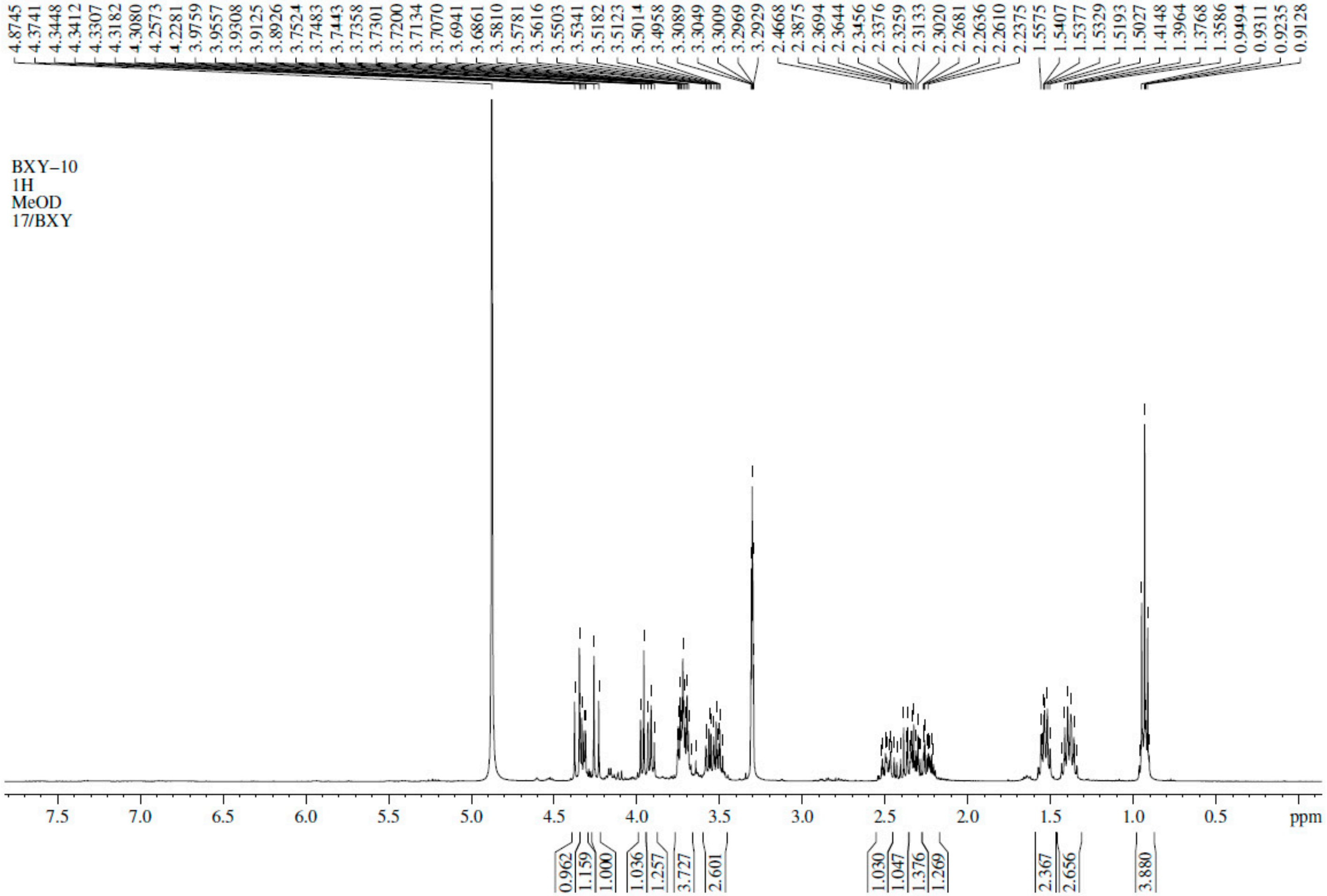

Fig.S2. ^{13}C-NMR Spectrum of Compound **1**

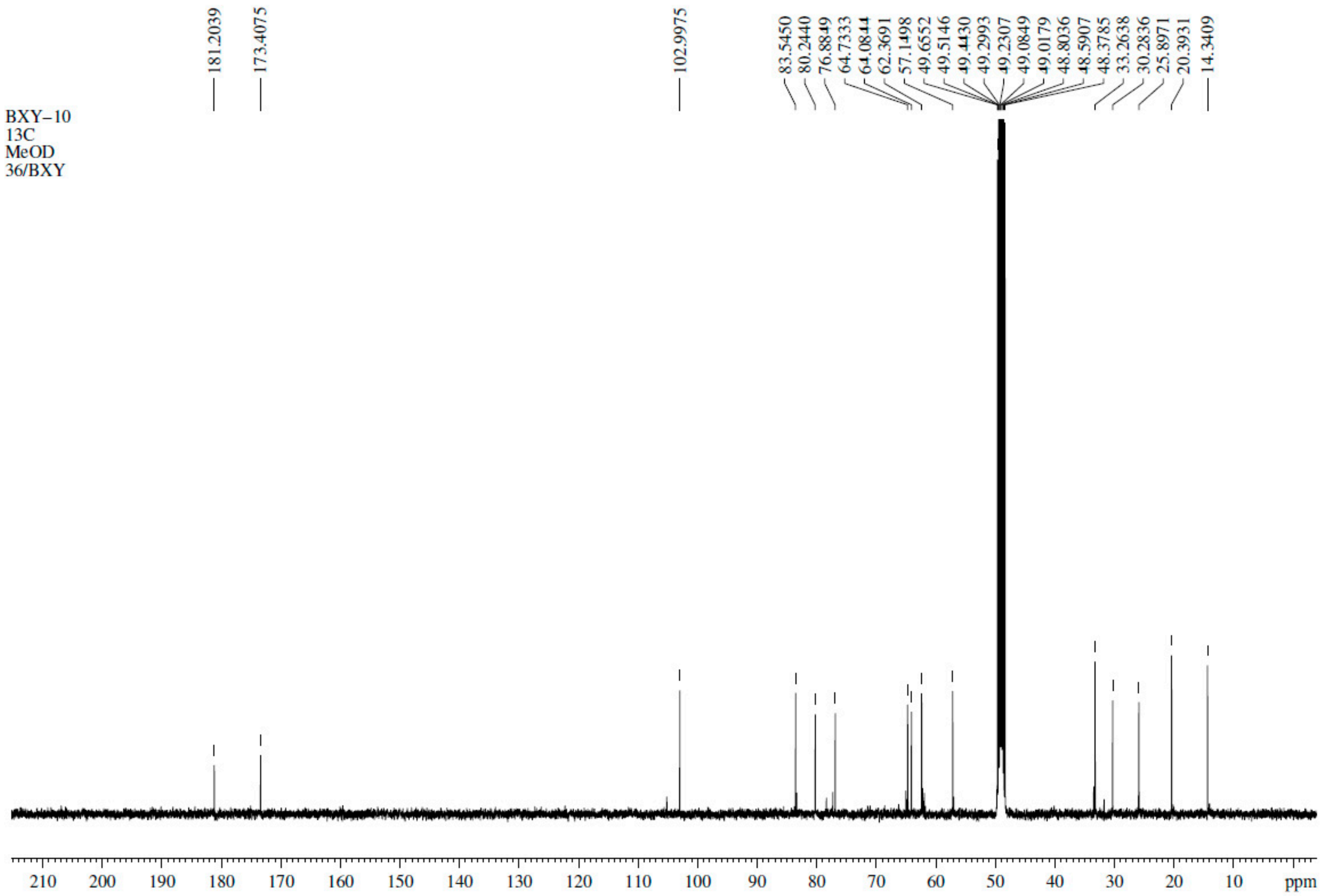

Fig.S3. ^{1}H-NMR Spectrum of Compound **2**

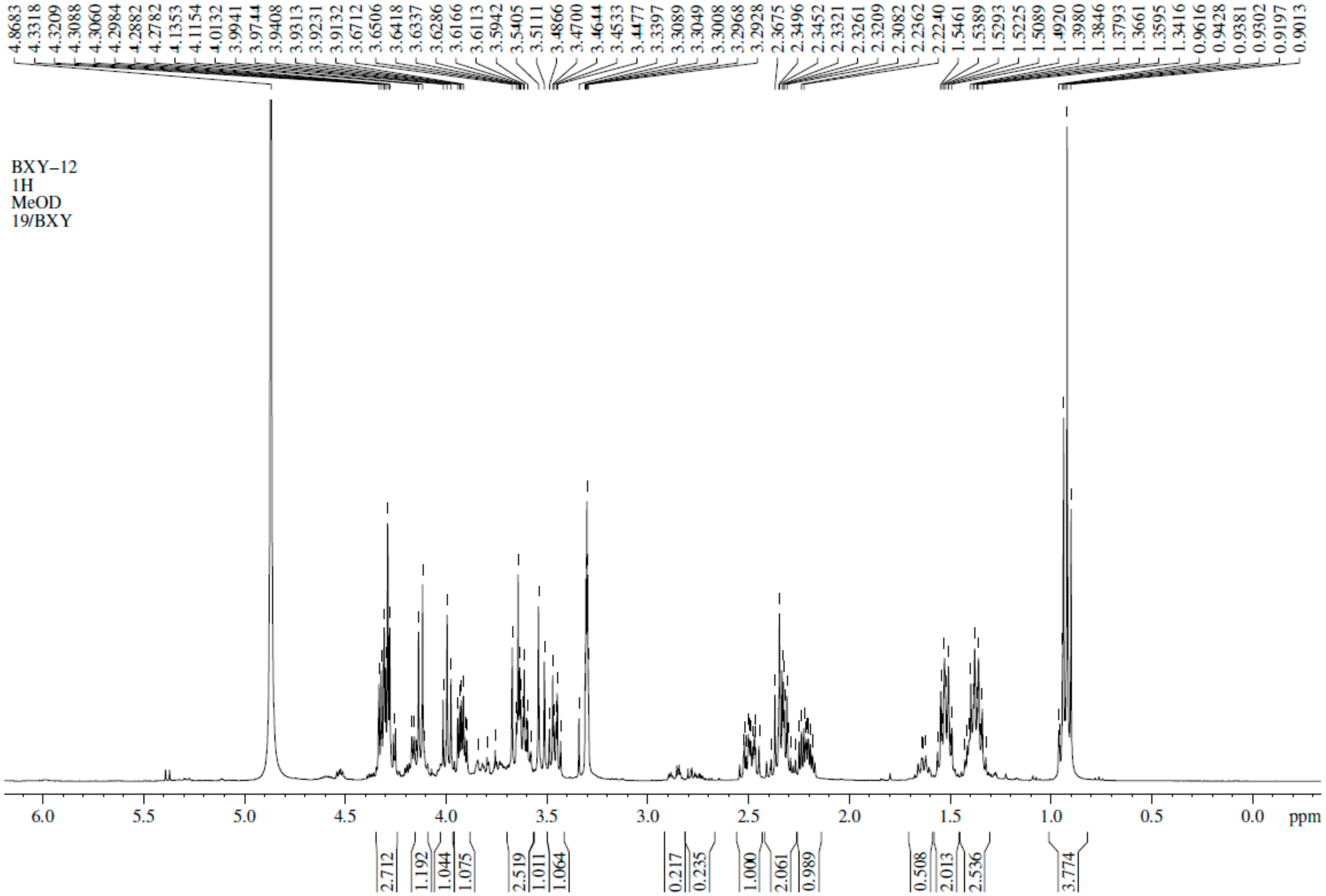

Fig.S4. ^{13}C-NMR Spectrum of Compound **2**

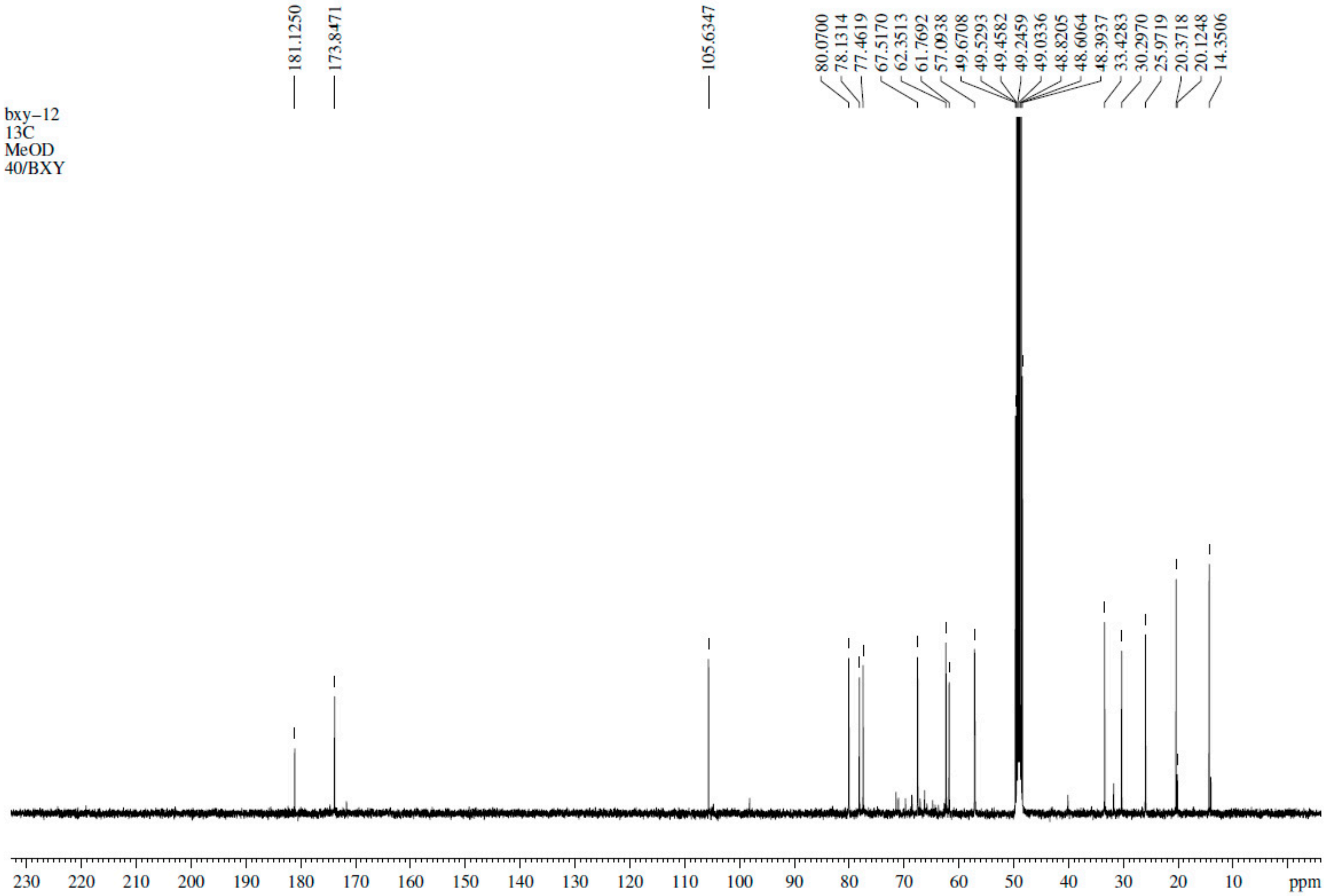

Fig.S5. ¹H-NMR Spectrum of Compound **3**

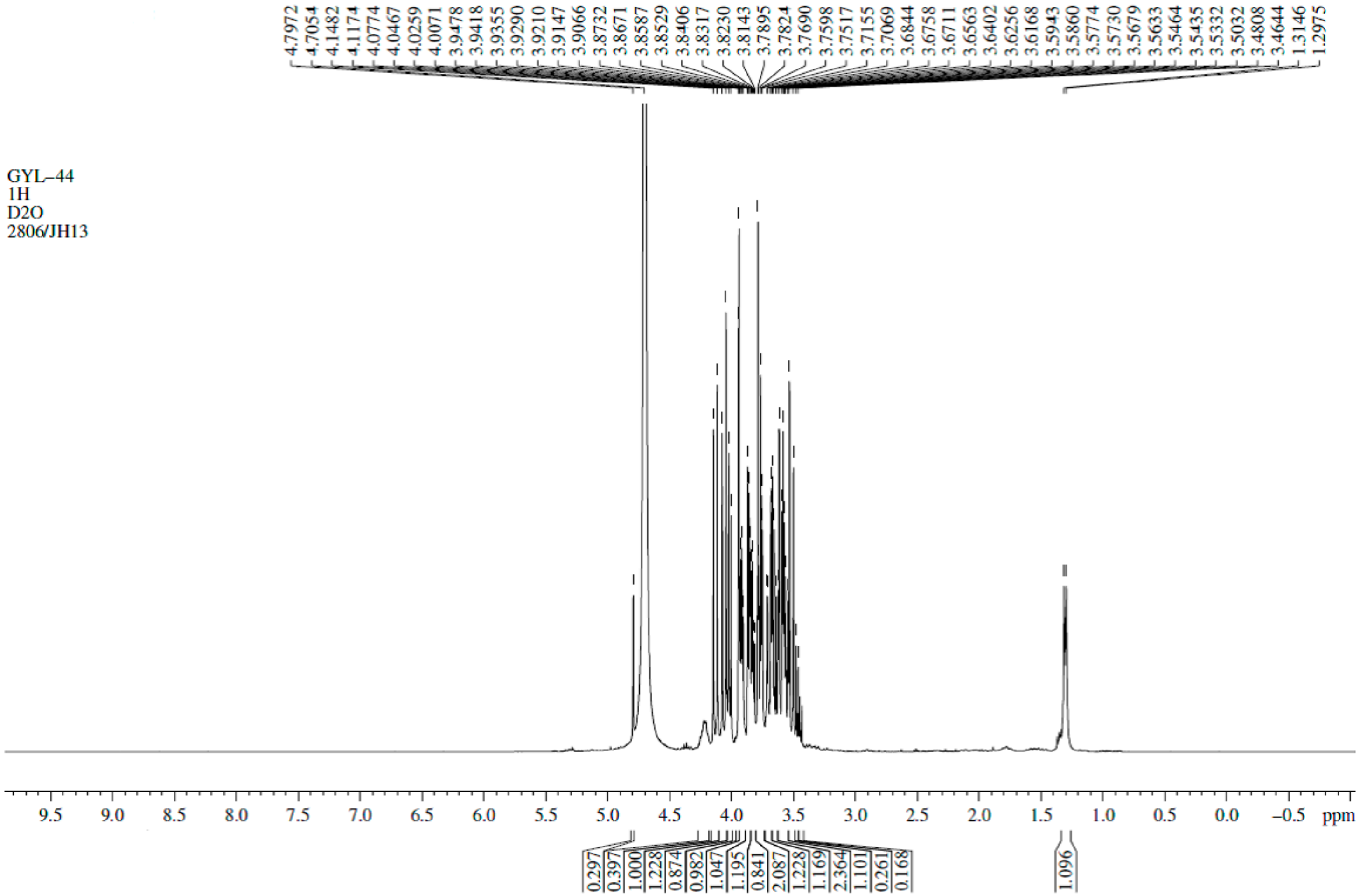

Fig.S6. ^{13}C-NMR Spectrum of Compound **3**

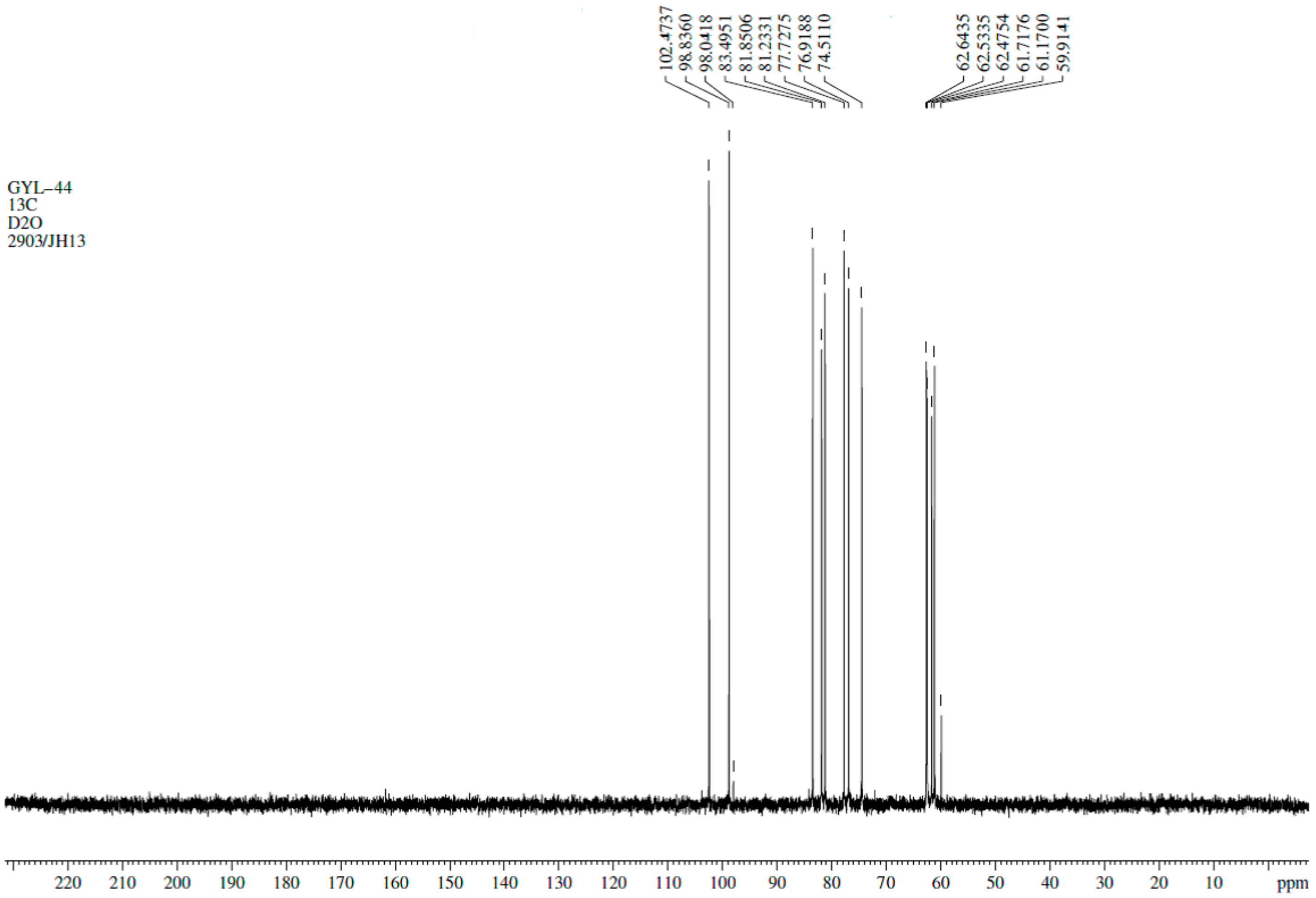

Fig.S7. ^{1}H-NMR Spectrum of Compound **4**

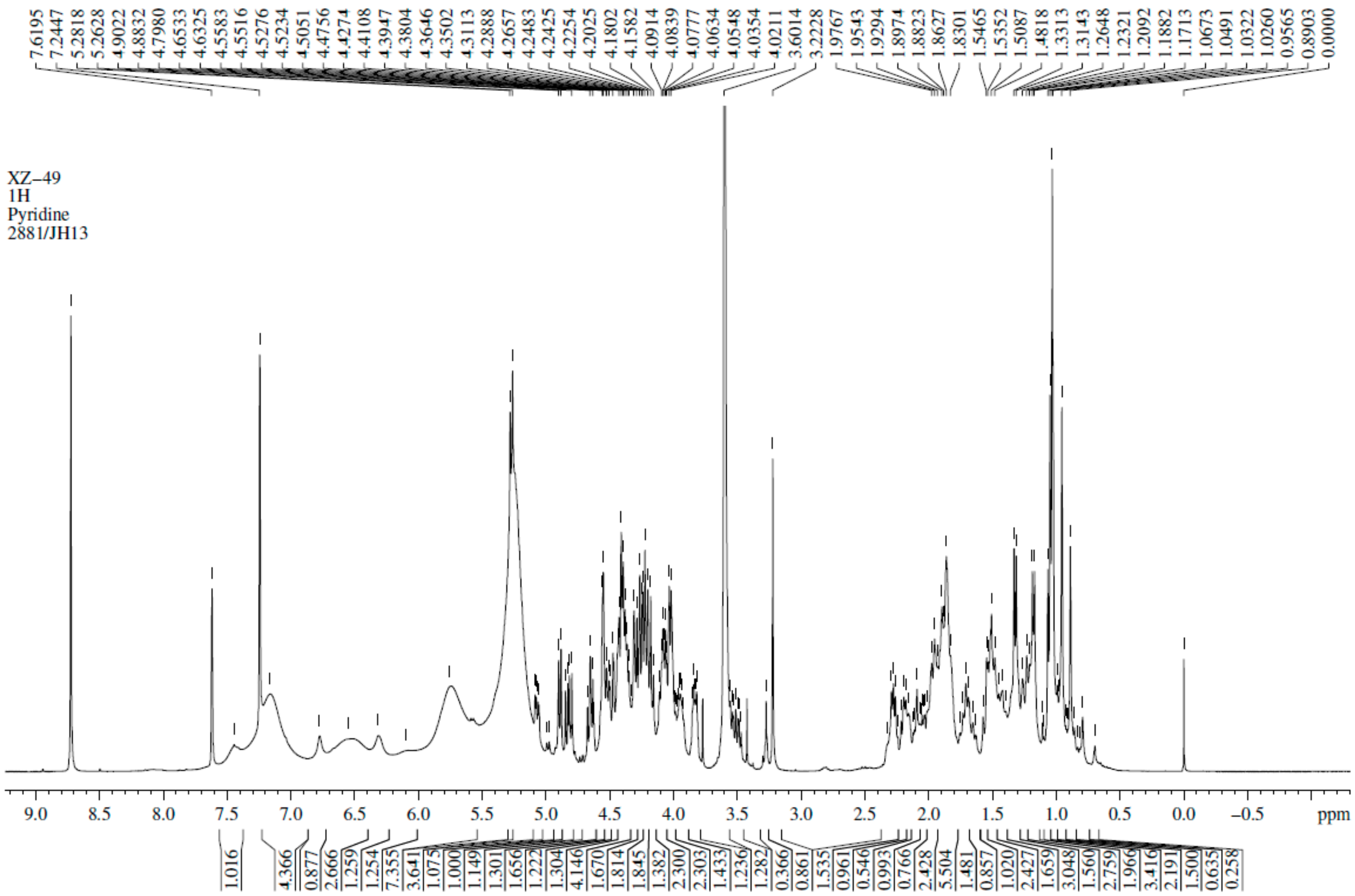

Fig.S8. ^{13}C-NMR Spectrum of Compound **4**

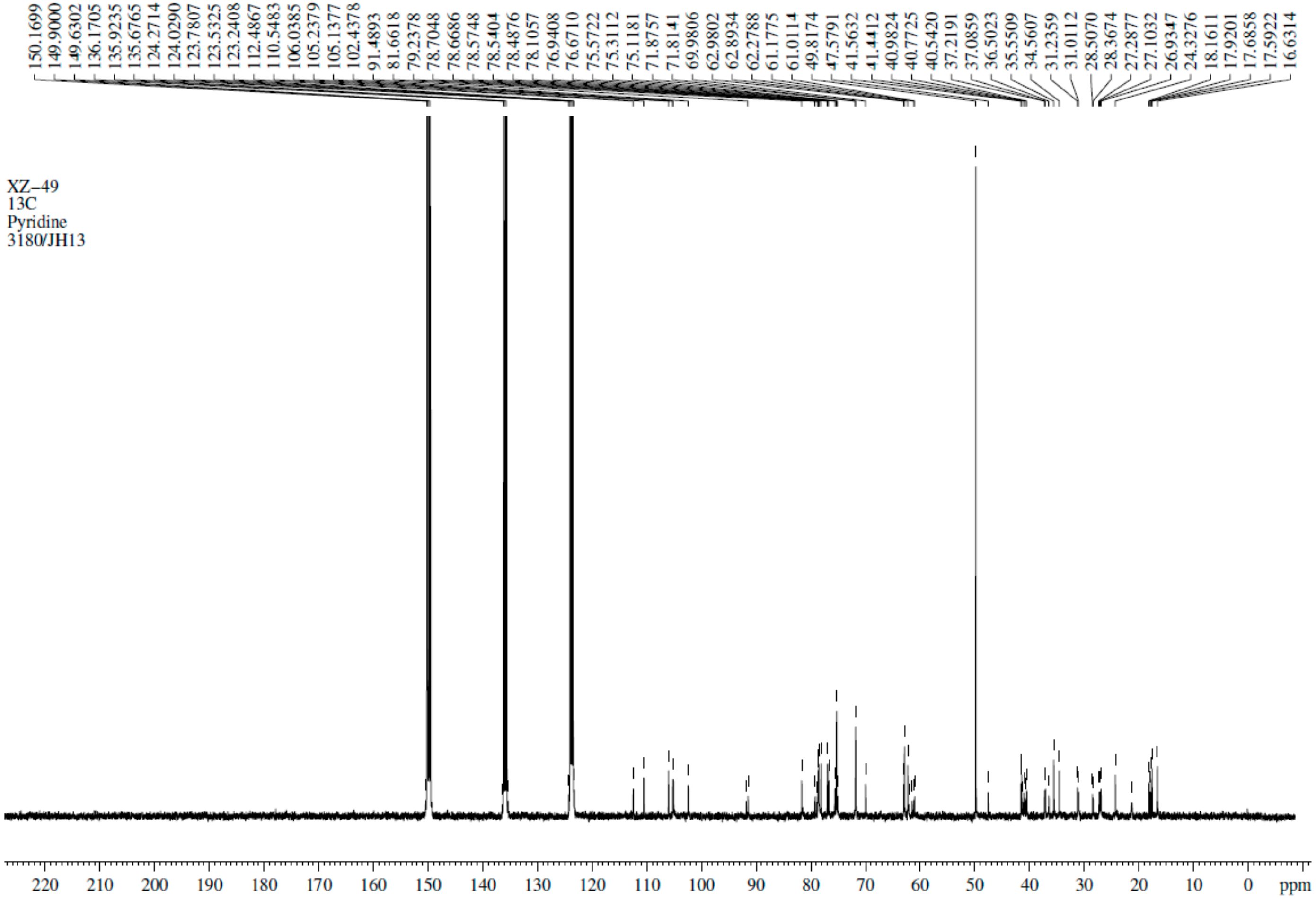

Supplement: Supplementary file 1 [file molecules-22-01995-s001.pdf]
